# Machine learning integration of multimodal data identifies key features of blood pressure regulation

**DOI:** 10.1016/j.ebiom.2022.104243

**Published:** 2022-09-06

**Authors:** Panayiotis Louca, Tran Quoc Bao Tran, Clea du Toit, Paraskevi Christofidou, Tim D. Spector, Massimo Mangino, Karsten Suhre, Sandosh Padmanabhan, Cristina Menni

**Affiliations:** a Department of Twin Research and Genetic Epidemiology, King's College London, London, England, SE1 7EH, United Kingdom; b Institute of Cardiovascular & Medical Sciences, University of Glasgow, Glasgow G12 8QQ, United Kingdom; c NIHR Biomedical Research Centre at Guy's and St Thomas’ Foundation Trust, London, SE1 9RT, United Kingdom; d Bioinformatics Core, Weill Cornell Medicine-Qatar, Doha, Qatar; e Department of Physiology and Biophysics, Weill Cornell Medicine, New York, NY, USA

**Keywords:** Blood pressure, Machine learning, Genomics, Metabolomics, Diet

## Abstract

**Background:**

Association studies have identified several biomarkers for blood pressure and hypertension, but a thorough understanding of their mutual dependencies is lacking. By integrating two different high-throughput datasets, biochemical and dietary data, we aim to understand the multifactorial contributors of blood pressure (BP).

**Methods:**

We included 4,863 participants from TwinsUK with concurrent BP, metabolomics, genomics, biochemical measures, and dietary data. We used 5-fold cross-validation with the machine learning XGBoost algorithm to identify features of importance in context of one another in TwinsUK (80% training, 20% test). The features tested in TwinsUK were then probed using the same algorithm in an independent dataset of 2,807 individuals from the Qatari Biobank (QBB).

**Findings:**

Our model explained 39·2% [4·5%, MAE:11·32 mmHg (95%CI, +/- 0·65)] of the variance in systolic BP (SBP) in TwinsUK. Of the top 50 features, the most influential non-demographic variables were dihomo-linolenate, cis-4-decenoyl carnitine, lactate, chloride, urate, and creatinine along with dietary intakes of total, trans and saturated fat. We also highlight the incremental value of each included dimension. Furthermore, we replicated our model in the QBB [SBP variance explained = 45·2% (13·39%)] cohort and 30 of the top 50 features overlapped between cohorts.

**Interpretation:**

We show that an integrated analysis of omics, biochemical and dietary data improves our understanding of their in-between relationships and expands the range of potential biomarkers for blood pressure. Our results point to potentially key biological pathways to be prioritised for mechanistic studies.

**Funding:**

Chronic Disease Research Foundation, Medical Research Council, Wellcome Trust, Qatar Foundation.

**Research in context**

**Evidence before this study**

Blood pressure is a complex polygenic multifactorial trait that is determined by a multitude of genetic, molecular, and physiological pathways interacting with one another. Although office blood pressure is highly variable, evidence from clinical trials conducted over the last five decades provide unequivocal evidence that higher blood pressure levels from either office or out-of-office measurements are highly predictive of early cardiovascular events and mortality. Understanding the mechanisms that regulate blood pressure so far comes from genetic and physiologic studies and this has led to effective treatment for hypertension. Omics studies hold the promise of identifying novel biological pathways that can lead to novel therapies. Genomic, metabolomic and other high-throughput technologies have allowed generation of valuable data and hypothesis generating insights, but the challenge for multifactorial traits such as blood pressure is the integration of several dimensions of data including multi-omics for discovery studies that truly represents the physiology of BP regulation. Machine learning allows for data from multiple sources to be integrated without many underlying assumptions and potentially offers an opportunity to derive insights from multimodal data. Previous studies that have attempted this have been limited by small sample size, lack of replication, or are restricted because they focused on a limited number of domains.

A study on 434 participants from the Finnish Twin cohort has integrated transcriptomic, methylation, clinical, metabolomics, and four clinical polygenic risk scores (for SBP, DBP, BMI and coronary artery disease) using the multi-block partial least square regression models and identified that a clinical polygenic risk score contributed to BP variability most, followed closely by metabolomics. Zheng and Yu (2021) integrated 12 clinical and lifestyle features in 500 participants, showing that the ML model could predict blood pressure to the highest standard set by governing bodies, including the British Hypertension Society.

**Added value of this study**

In the most comprehensive study to date, we have applied ML on multimodal domains covering environmental, dietary, genetics, metabolites, biochemical, and clinical data and identified the key features contributing to BP regulation. We have demonstrated the value of ML in dissecting blood pressure. The list of the most important features identified will trigger future studies to build on our model, validate those features, and identify novel pathways that may be targetable by drugs.

**Implications of all the available evidence**

We have extended this body of work by applying ML and big data from multiple domains and we provide an incremental advance of our current understanding of blood pressure regulation. The results from this study align with and extend other models. For instance, our study integrated 264 variables in a ML model to identify the top 50 variables that influence blood pressure. We speculate that this set of variables may provide the foundation for future study designs considering a minimal informative set of variables to be included. Our top variables will inform validation studies followed by investigation of underpinning pathways.

The results of our study are early in the clinical translation runway. They provide an incremental advance to prioritise certain metabolic pathways in conjunction with diet and biochemical pathways. The next step would be to focus on these pathways to identify underpinning mechanisms.

Alt-text: Unlabelled box

## Introduction

Hypertension, defined as high blood pressure (BP), is the leading modifiable risk factor for cardiovascular disease, affecting >1·5 billion adults globally.^1^

BP is a complex multifactorial phenotype involving a multitude of physiological pathways in conjunction with genomic, demographic, lifestyle, and environmental factors.^2^, ^3^, ^4^ BP levels above a threshold are termed hypertension, however, this is a false dichotomy as cardiovascular risk increases with every mmHg increase in BP from 115 mmHg systolic, with clinical guidelines progressively reducing the threshold for hypertension treatment since the 1970s.^5^ Discovering causal pathways that determine blood pressure and its dysregulation has resulted in effective pharmacotherapy and public health policies to reduce the burden of hypertension.^6^ This includes the recognition of numerous socio-demographic and risk factors, such as, educational status,^7^ and race,^8^ and research into aldosterone receptors, which resulted in the development of dihydropyridine calcium channel blockers, a drug with profound effects on BP.^9^ However, the pace of progress has faltered over the last 2 decades with no new drugs licensed for hypertension and a plateauing of the rate of hypertension control achieved worldwide.^10^, ^11^, ^12^ This may partially be explained by the limitation of underpinning studies that hitherto informed prevention and treatment of hypertension that were based on the investigation of single mechanistic pathways,^13^ described in greater detail in.^2^ Recent advances in high-throughput technologies that allows detailed data on different biological systems to be generated, along with newer analytic methods including machine learning (ML), opens-up opportunities to conduct integrated analyses of hypertension that truly captures its underlying complexity. Thus allowing for new insights to be generated in the drive for new drug development or diagnostic/preventive applications. Drouard and colleagues^14^ integrated genetic, methylation, transcriptional, and metabolomic data in a cross-sectional cohort and found a clinical polygenic risk score to be the most important dimension for predicting BP, followed closely by metabolomics. While Zheng and Yu^15^ have shown that a ML model built on clinical and lifestyle features can predict BP with a low margin of error.

ML can be applied to every aspect of the human condition.^16^ In contrast to commonly employed statistical techniques, ML algorithms are powerful tools with the capacity to integrate multimodal data, typically without making many assumptions of the underlying data and their applications in hypertension research is growing.^17^ Decision trees in particular are a supervised ML method with the capacity to rank the input features based upon their relative importance on the outcome.^18^

In this large cross-sectional study, we applied the decision tree-based machine learning method Extreme Gradient Boosting (XGBoost) to improve our understanding of the multifactorial contributors of BP regulation in context of one another by identifying the top BP contributors. We integrated multimodal data (metabolomics, genomics, biochemical, and dietary data) from the deeply phenotyped TwinsUK cohort and validated our results in the Qatari biobank (QBB) cohort.

## Methods

### Study participants

Discovery: Our discovery dataset was comprised of twins enrolled in the TwinsUK registry, a national register of adult twins recruited as volunteers without selecting for any particular disease or traits.^19^ This sample included 4,863 participants (1,113 monozygotic twin pairs, 1,136 dizygotic twin pairs and 365 singletons) aged between 17 and 75 years, who were not on any BP-lowering treatments, with concurrent measurements for metabolomics assessed by Metabolon (Metabolon Inc, Durham, USA), and genomics available as well as phenotypic information including age, BMI, sex, electrolytes and estimated dietary intake from food frequency questionnaires (FFQs).

Replication cohort: We then tested model performance, trained in TwinsUK, in an independent sample from the QBB. The QBB is a prospective, population-based cross-sectional cohort in Qatar. QBB was established to investigate a host of health-related questions through evidence-based research and described in detail in.^20^^,^^21^ Here, we included 2,807 individuals who had blood pressure data as well as metabolomics assessed by Metabolon (Metabolon Inc, Durham, USA), and concurrent phenotypic information including age, BMI, sex, and electrolytes. FFQs were not available.

### Data acquisition and processing

The phenotypic data was collected using questionnaires and anthropometric measures taken during visits to a clinical research facility.

### Metabolomics

Samples were collected after an overnight fast for TwinsUK, while QBB participants were non-fasted. Circulating metabolite levels were measured from plasma and serum samples using an untargeted LC/MS and GC/MS platform by Metabolon Inc., Durham, USA as previously described.^22^^,^^23^ Briefly, proteins were precipitated and chemically diverse metabolites were isolated with methanol under vigorous shaking for 2 minutes followed by centrifugation. The resulting extract was divided into four fractions: one for analysis by ultra-high performance liquid chromatography-tandem mass spectrometry (UPLC-MS/MS; positive mode), one for analysis by UPLC-MS/MS (negative mode), one for analysis by gas chromatography–mass spectrometry (GC-MS), and one sample was reserved for backup. Three types of controls were analysed in concert with the experimental samples: samples generated from a pool of human plasma (extensively characterized by Metabolon, Inc.) served as technical replicates throughout the data set; extracted water samples served as process blanks; and a cocktail of standards spiked into every analysed sample allowed instrument performance monitoring. Experimental samples and controls were randomised across the platform run.

The UPLC-MS/MS platform utilized a Waters Acquity UPLC and a ThermoFisher LTQ mass spectrometer, which included an electrospray ionization source and a linear ion-trap mass analyser. The instrument was set to scan 99-1000 m/z and alternated between MS and MS/MS scans. The instrumentation was set to monitor for positive ions in acidic extracts or negative ions in basic extracts through independent injections

All samples were analysed on a Thermo-Finnigan Trace DSQ MS operated at unit mass resolving power with electron impact ionization and a 50-750 atomic mass unit scan range. Metabolites were identified by automated comparison of the ion features in the experimental samples to a reference library of chemical standard entries that included retention time, molecular weight (m/z), preferred adducts, and in-source fragments as well as associated MS spectra and curated by visual inspection for quality control using software developed at Metabolon.^24^ Identification of structurally named chemical entities is based on comparison to a mass spectroscopy library of >2,400 purified standards. Peaks were quantified using area under the curve.

Only known metabolites were included and they belonged to the following major classes - amino-acids, peptides, carbohydrates, energy intermediates, lipids, nucleotides, cofactors and vitamins, and xenobiotics.

### Genomics

Genomic sequencing data was generated from blood samples taken during a clinical visit using the Illumina HumanHap300 BeadChip and Illumina HumanHap610 QuadChip (Illumina, Cambridge, UK). Non ­genotyped variants were then imputed using the 1000 Genomes reference panels. Quality control was performed by validating pooling by visually inspecting 100 random, shared Single Nucleotide Polymorphisms (SNPs) for overt batch effects, and visually checking for erroneous genotype assignment using intensity cluster plots of significant SNPs. SNPs exhibiting any of these characteristics were discarded.

891 SNPs from the large BP genome-wide association study (GWAS) conducted by Warren and collaborators^25^ were included and the polygenic risk score (PRS) for BP risk was calculated by summing an individual's risk alleles, which were weighted by effect sizes derived from GWAS data.^25^^,^^26^

### Clinical phenotypes

#### Blood pressure

BP was measured by a trained nurse and performed with the patient in the sitting position for 3 minutes as previously described.^27^ The cuff was placed on the subject's arm so that it was approximately 2-3 cm above the elbow joint of the inner arm, with the air tube lying over the brachial artery. The subject's arm was placed on the table or supported with the palm facing upwards, so that the tab of the cuff was placed at the same level of the heart. Triplicate measurements were taken with an interval of approximately 1 minute between each reading, with the mean of the second and third measurements recorded.

Besides omics data and BP measurements, data relevant to the present study included biochemical measures [sodium, bicarbonate, potassium, and chloride, measured using the Kodak Ektachem dry chemistry analyser^21^^,^^28^].

#### Diet

FFQs based upon the EPIC FFQ,^29^ an FFQ validated against biomarkers in the European Prospective Investigation into Diet and Cancer Norfolk (EPIC) were used to estimate dietary intake.^29^^,^^30^ FFQs were then coded and processed using FETA,^31^ an open-source, cross-platform tool designed to process dietary data from the EPIC FFQ, in accordance with their guidelines. Intakes for 45 nutrients and energy intake were then estimated by the software and adjusted for energy intake using the residual method.^32^ We included FFQs data that were on average 2.5 (3.1) years from the BP measurement.

## Statistical analysis

**Figure 1:**
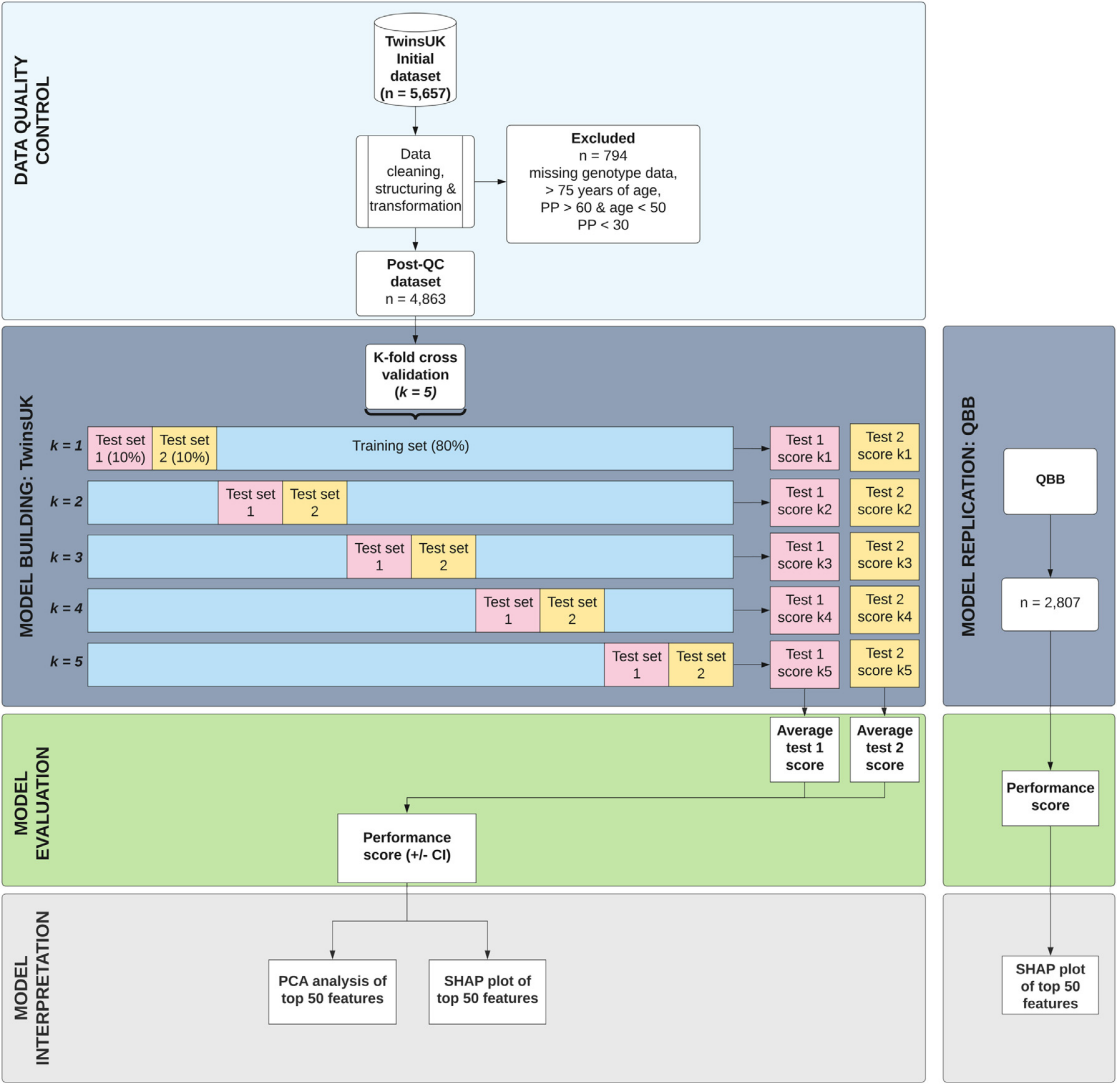
**Consort diagram of data quality control, machine learning model building and model evaluation.** Data included traditional risk factors (age, sex and BMI), biochemical measures, 206 known metabolites, a BP polygenic risk score, and energy intake and dietary intake for 45 nutrients (including salt intake).

A flowchart of the study design is presented in Figure 1.

### Data quality control

The original raw dataset consisted of 5,657 individuals (Figure 1). As with ML algorithms missing data reduces statistical power, introduces bias in the estimation of parameters and decreases the representativeness of the samples,^33^ we imputed the missing data across different omics datasets as detailed below.

*Genotypes:* missing genotype data was imputed from a twin in a monozygotic twin pair using the genotype data of the other twin. The remaining individuals with missing genotype data were excluded.
*Metabolites:* Quality control of metabolomics data was carried out as previously described. Briefly, metabolite concentrations were inverse normalised using a Rank-based inverse normal transformation^34^ to counteract abnormal distribution. To avoid spurious false-positive associations because of a small sample size, metabolic traits with >20% missing values were excluded. Missing values were then imputed using the minimum run day measures.
*Phenotypes:* Any traits with >20% missing values were excluded. The remaining missing values were imputed using the K nearest neighbour's imputation algorithm (KNN), which uses the weighted average from the nearest neighbours of the sample.

As the BP of patients with narrow or widened pulse pressure was likely to be influenced by an underlying condition^35^ the dataset was further filtered based on the following exclusion criteria: >75 years of age, if age < 50 years & pulse pressure > 60 or pulse pressure < 30 (Figure 1). The final dataset of 4,863 patients was then used for several pre-analysis tasks, including encoding categorical data and predictors into numerical values, and feature selection (see Figure 1).

### Machine learning model

We constructed ML models for SBP and DBP separately. A total of 264 features were included for the construction of the ML models: metabolites: 206 circulating metabolites of known biochemical identity; genetics: the polygenic risk score for BP risk; dietary data: energy intake and intake of 45 nutrients and biochemical measures: alongside the traditional risk factors, age, BMI, and sex. To account for family relatedness and clustered effects, one variable indicating a unique identifier per family and metabolite batches in the input data was included. The inclusion of cluster IDs as candidates in the splitting process to adjust for clustered effect in decision tree-based model has been proven to be practical and unlikely to distort the results.^36^^,^^37^

Traditional linear regression models often fail when input features have a non-linear relationship with the outcome, or when there are interactions between the features. To better capture the complex mechanisms underlying blood pressure control by multiple biological factors, the decision tree-based XGBoost algorithm was employed. The XGBoost algorithm has been successfully used for a wide range of medical applications, including disease diagnosis, survival estimation, outcomes, prognosis, drug research and development.^38^

XGBoost was created by Chen and Guestrin^39^ as an ensemble of multiple decision trees. Decision trees are a robust ML model capable of a high degree of accuracy and interpretability.^39^^,^^40^ They start with a root node, which contains all the features included in the training data. The root node is then split into multiple smaller nodes, each containing a subset of the features. The decision on when to make a split is based on the reduction of variance in the child node compared to the parent node after a split.^41^

This process continues recursively until the variance in a node reaches zero or there are no more features for splitting. The final node where further splitting is no longer feasible is referred to as a leaf or a terminal node. Afterwards, the resulting model can predict the outcome for a new observation based on its covariates by determining which terminal node it belongs to.

The XGBoost algorithm generates a sequence of weak decision tree models, as above, with every subsequent tree aiming to correct the errors made by the previous tree. XGBoost does so by recursively fitting new models to the residuals of the previous models. Essentially, each new tree in the sequence will focus on minimising the errors of the previous tree. As a result, the final ensemble model will have better performance than its base tree models. Accordingly, when hyperparameters are tuned appropriately (hyperparameter tuning in this study is shown in Supplementary Table 1), XGBoost is perfectly suited to investigate and rank the most important features involved in a health outcome, such as blood pressure regulation.

### Model evaluation

Here, we used a hypothesis generating approach and did not apply any prior knowledge to feature selection. The performance of the model is evaluated using 5-fold cross-validation. This split ratio is based on the Pareto Principle (80/20 rule),^42^ which specifies that 80% of outcomes are derived from 20% of causes. In the standard cross-validation approach, the training set is split into 5 smaller sets. The model is trained using 4 sets (80% of original dataset) and is validated using the remaining set (20% of original dataset). This process is then repeated 4 more times, each time with a different test and training set (Figure 1).

However, in this approach, the splitting of the dataset is entirely random. As such, one twin may be included in the training set and the other twin in the testing set. This may lead to data leakage, resulting in overly optimistic results. Hence, Sklearn's GroupShuffleSplit method was employed in the present study^43^^,^^44^ to constrain the splitting process of each fold with the family ID, such that every twin pair will always stay together in either the training or testing set. To prevent double counting, the test set of each fold was further split into 2 smaller sets (namely, a test 1 and test 2), with each twin of a pair being randomly allocated into one set. The final performance measure reported by the cross-validation is the average of the values computed in all 5 folds (Figure 1).

### SHapley Additive exPlanation (SHAP) values

To further understand and interpret the features within our model, we used SHapley Additive exPlanation (SHAP)^45^ values to determine feature importance and visualise the inner workings of our ML model. The SHAP method was developed by Scott Lundberg and Su-In Lee^45^ based on the concept of game theory. SHAP values can be calculated by individually adding features to the feature set and checking the change in model output accordingly to determine a feature's relevance to the final prediction. In other words, the SHAP value can be rationalised as the average of the marginal contributions across all permutations, given a particular model output. SHAP values have an explicative role, and they quantify the magnitude of contribution (feature importance) as well as the direction (positive or negative) of a feature's effect on a prediction, which can then be used to explain each of the features’ role on the prediction of the ML model. SHAP values from all 5 cross-validation folds were pooled to construct the final SHAP value set. In this study, we pruned the input features to generate a manageable list of the top 50 features and the SHAP summary plot is depicted to identify the relative importance of these features.

To further interpret the most influential features of our ML model and understand their relative relationships to one another, we further conducted a principal components analysis (PCA) in the top 50 features and visualised the output in the form of a biplot using the python library sklearn.^43^ We then investigated the roles of the top 50 features from our algorithm in various biological pathways as implemented in the Ingenuity Pathway Analysis (IPA) database^46^ (QIAGEN Inc, https://www.qiagenbioinformatics.com/products/ingenuity-pathway-analysis). IPA pathway analysis connects our top 50 features which are likely to be part of the same signalling or causal mechanism in hypothesis networks.

### Replication in QBB

The XGBoost model was then used again in the QBB cohort integrating the same input data as was used in TwinsUK. The model was deployed utilising the Linux-based Docker package.^47^ We then used a SHAP summary plot to visualise the top 50 features influencing SBP in the QBB cohort.

### Ethics

In accordance with the declaration of Helsinki, all participants provided informed written consent. The TwinsUK study was approved by St. Thomas’ Hospital Research Ethics Committee (REC Ref: EC04/015). The Qatar Biobank study was approved by Hamad Medical Corporation Ethics Committee and Qatar Biobank institutional review board. Use of the Qatar Biobank data was approved under reference Ex -2019-RES-ACC-0160-0083.

### Role of the funding source

The funding sources had no role in the study design, collection of data, analysis, or interpretation of data, writing of the manuscript or the decision to submit for publication.

## Results

### Demographics

The demographic characteristics of the study populations are included in Table 1. Briefly, the discovery cohort comprised 4,863 individuals (92·8% female) who were not using antihypertensive treatments. The mean age of the cohort was 53·46 (±13.2) years, and on average they were marginally overweight with a mean BMI of 26·15 (±4·9) kg/m^2^ and normotensive (SBP: 127·42 (±18) mmHg and DBP: 77·24 (±10.1) mmHg).

**Table 1:** Demographic characteristics of the study population.

Phenotype	TwinsUK		QBB		
n	4,863		2,807		
Female, n (%)	4513 (92.8%)		1,398 (49.8%)		
	***Mean***	***SD***	***Mean***	***SD***	***P***
Age, yrs	53.46	13.2	39.11	12.0	8.06 × 10^−14^
BMI, kg/m2	26.15	4.9	28.92	5.9	3.88 × 10^−4^
SBP, mmHg	127.42	18	114.6	15.2	1.59 × 10^−7^
DBP, mmHg	77.24	10.1	73.29	10.5	7.29 × 10^−3^
Creatinine, µmolL	73.24	22.06	67.67	19.7	0.06
Macronutrient intake*:					
Energy, Kj	7991.46	2316			
Carbohydrates, g	238.91	77.4			
Protein, g	80.37	22.9			
Total fat, g	68.01	25			
*Electrolytes:*					
Chloride, mmol/L	103	3.19	101.1	2.16	1.89 × 10^−6^
Sodium, mmol/L	140.61	2.38	140.3	2.19	0.34
Potassium, mmol/L	4.16	0.32	4.32	0.32	5.07 × 10^−4^
Bicarbonate, mmol/L	25.52	2.45	26.39	2.02	6.73 × 10^−3^
Calcium, mmol/L	2.37	0.1	2.39	0.092	0.14
Phosphate, mmol/L	1.11	0.16	1.14	0.171	0.20

*Prior to adjustments for energy intake.

The QBB cohort, on the other hand, was 49.8% female, younger, with an average age of 39.1 (±12) years, had an average BMI of 28.92 (±5.9) kg/m^2^ and were also normotensive (SBP: 114.6 ±15.2, DBP: 73.29 ±10.5).

### Feature importance in TwinsUK

**Figure 2:**
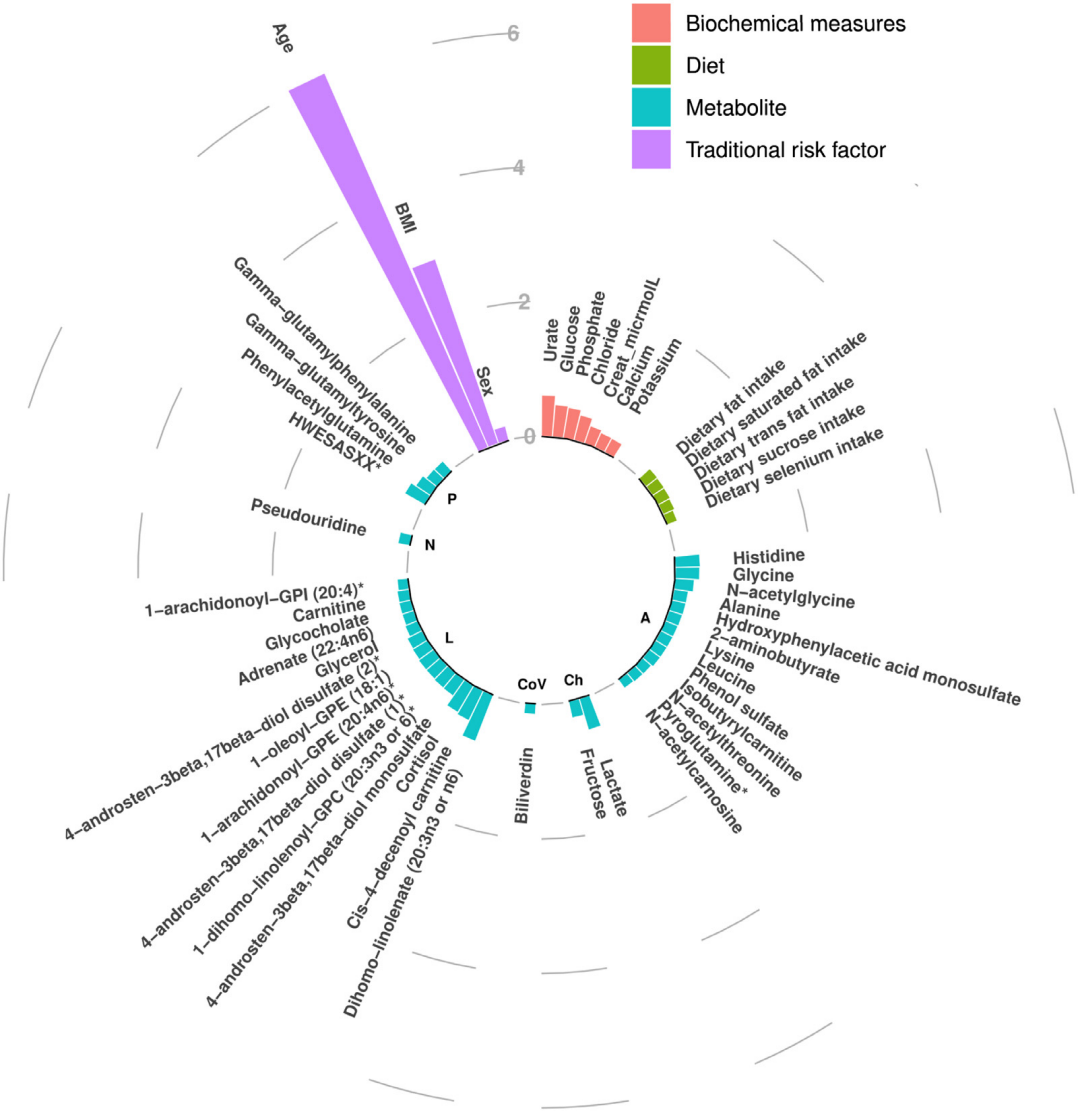
**Importance of top 50 features in SBP**. Bars represent SHAP values indicating the average relative importance of each feature, coloured according to the type of data. Base layer labels indicate metabolite super pathways, where; A=Amino Acids, Ch=Carbohydrates, L=Lipids, N=Nucleotides, P=Peptides, and CoV=Cofactors and vitamins.

The XGBoost ML algorithm was leveraged to identify the importance for each of the 264 features in BP regulation (the top 50 are shown in Figure 2). As expected, the traditional risk factors, age, and BMI were the features with the largest magnitude of effect on SBP and these were followed by 7 biochemical measures, 5 dietary variables, and 35 metabolites in the top 50 features. However, the PRS did not make the top 50 features. The feature importance plot for DBP is similar, with a large preponderance of metabolites, 16 of which overlapping with SBP.

**Figure 3:**
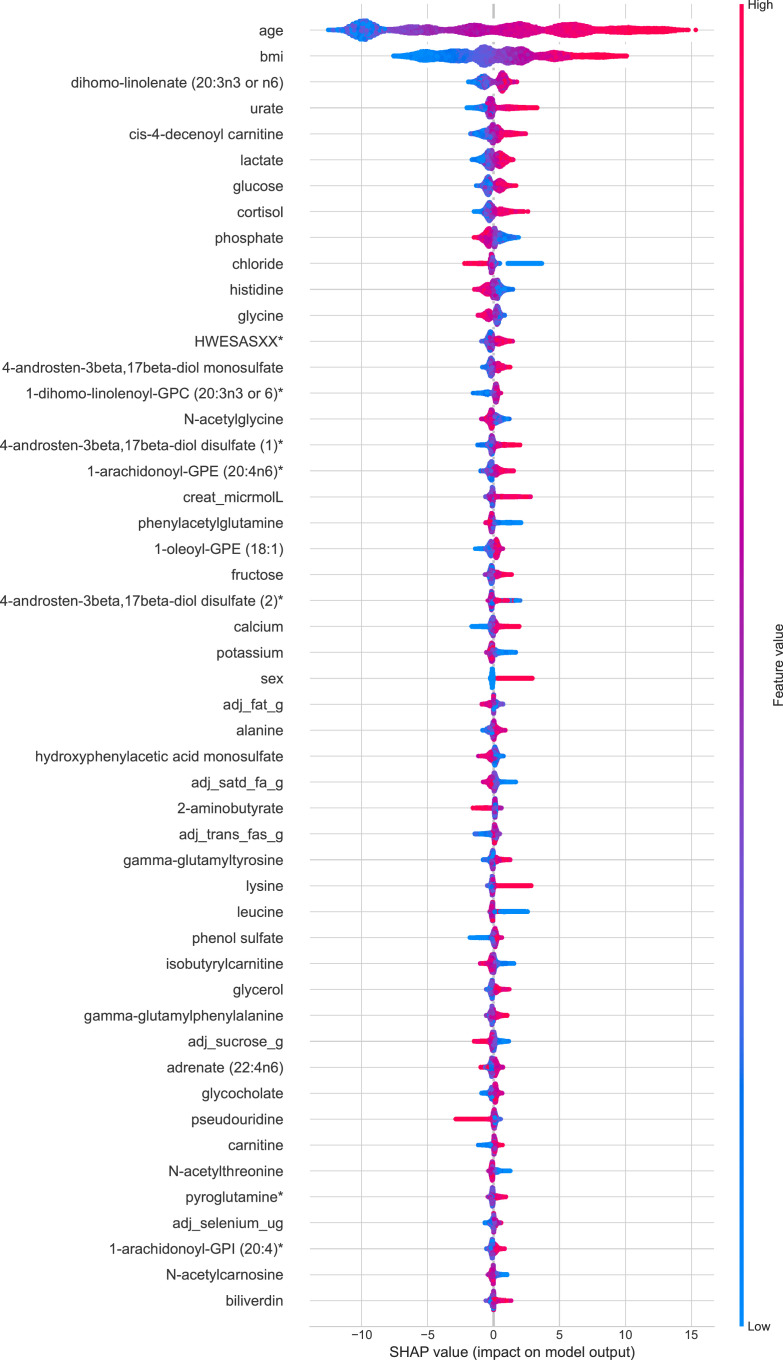
**SHAP plot of top 50 features influencing our model's prediction of SBP.** Features are ranked in descending order based on their influence on our model and the x-axis denotes SHAP values. Each dot represents an individual subject and coloured according to the magnitude of the feature (red depicts a higher feature value, and blue depicts a lower value). The horizontal location of a dot (x-axis) depicts whether it corresponds with a higher or lower prediction.

The SHAP summary plot visualises the SBP analysis using XGBoost (Figure 3) and delineates the top 50 features of the prediction model. Age and BMI ranked as the most important variables contributing to both SBP and DBP models. For SBP, of the 35 metabolites, the SHAP analysis identified dihomo-linolenate, cis-4-decenoyl carnitine, lactate, and cortisol as the most important, while urate, phosphate, chloride, and dietary fat intake ranked as some of the most influential features for electrolyte and dietary variables (Figure 3). Similarly, SHAP analysis of DBP features also highlights the influence of cis-4decenoyl carnitine, lactate, urate, dietary trans-fat intake and dihomo-linolenate (Supplementary Figure 1). The x-axis indicates the SHAP values of the top 50 features of importance, while the y-axis shows the features used in the model's predictions. The features are ranked in descending order, with the top feature having the highest influence on the model. For every feature, each dot represents an individual patient from the original dataset (n=4,863). Dots are coloured according to the magnitude of the features for the respective patients. Red depicts higher feature values, while blue depicts lower feature values. The horizontal location of a dot shows whether its corresponding feature value is associated with a higher or lower prediction. A feature with higher SHAP value contributes more towards higher BP prediction.

### Model performance in TwinsUK

5-fold cross-validation of the prediction model on the test set indicated a mean absolute error (MAE) of 11·32 mmHg (95% CI, +/- 0·65), mean absolute percentage error MA%E) of 8·92% (95% CI, +/- 0·51%), and R^2^ of 39·2% (95% CI, +/- 4·5%). A sensitivity analysis was also performed by excluding males from our dataset (350 male participants, 7·2%). Prediction estimates were consistent. The female-only model (n=4513) produced a MAE of 11·43 mmHg (95% CI, +/- 0·92), MA%E of 9·0% (95% CI, +/- 0·66%), and R2 of 33·5% (95% CI, +/- 8%). Our sample did not have enough male participants to conduct a male-only model. This marginal increase in variance in the female-only model is to be expected with a 7·2% reduction in sample size. Additionally, to determine if our ML algorithm can pick up subtle differences even between twins, we conducted a sensitivity analysis by removing the cluster id from our model. Results were consistent with only minor differences between the model with and without the clustering id (Supplementary Table 2). When exploring the added benefits of included dimensions, we found that our input features explained an extra 6% of the variance in BP over traditional risk factors (Supplementary Table 2). We also note that the addition of metabolites to biochemical measures and traditional risk factors brought about a 1% increase in R^2^, though the further addition of dietary features to biochemical measures, traditional risk factors, and metabolomic features only improved R^2^ by a further 0·1%.

**Figure 4:**
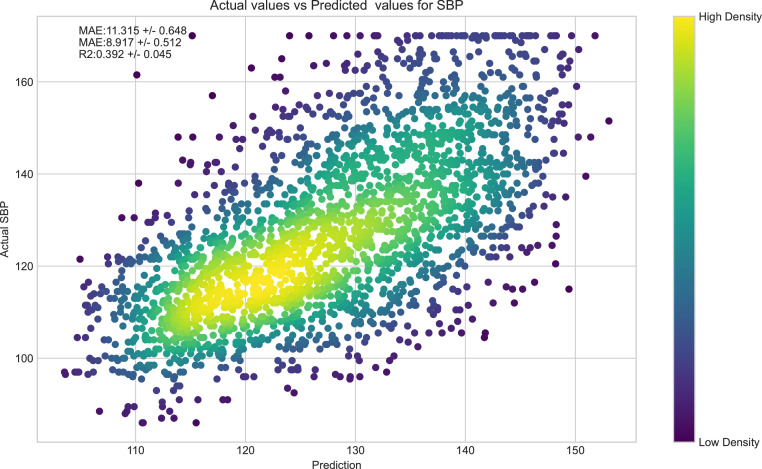
**Scatter plot of SBP within our sample and predicted SBP of the XGBoost algorithm.** Actual SBP of each subject within our sample plot along the y-axis and predicted SBP from our model across the x-axis (in mmHg). The colour gradient of each point denotes the density of participants within a particular region of the plot.

Figure 4 presents a scatter plot showing a relatively linear relationship between the actual SBP and the SBP generated by the algorithm, supporting the accuracy of our model to depict SBP from metabolomic, electrolytes, and other biochemistry data. The values in the plot are pooled from all 5 folds of the cross-validation process. However, the paucity of individuals with SBP>150 mmHg within our dataset limits our model's capacity to predict SBP values above this value (Figure 4).

**Figure 5:**
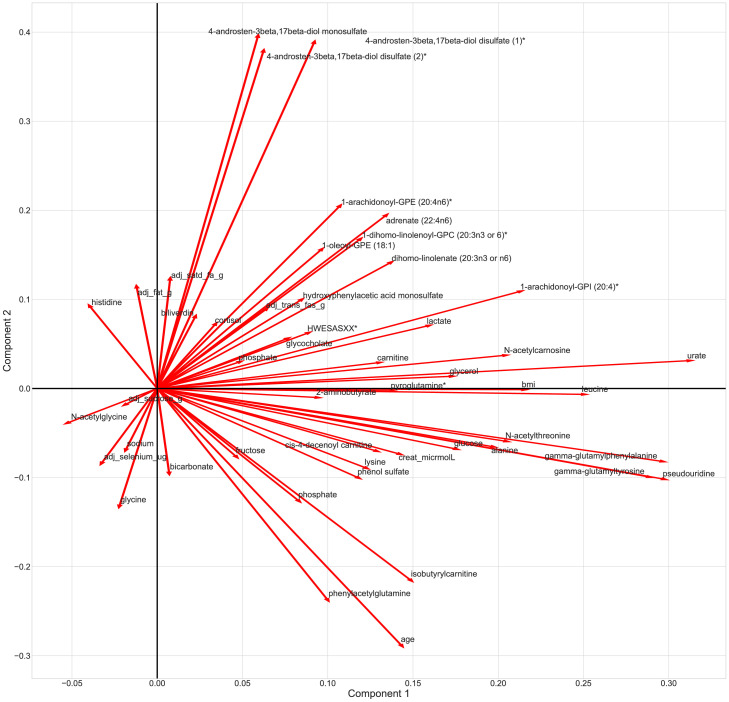
**Bi plot depicting the principal component analysis of features most influential to our SBP model.** Lines depict how strongly each feature influences a principal component, and the angle between each feature represents the correlations between those features.

We further employed PCA to identify plausible pathways and clusters/relationship of features involved in BP regulation (Figure 5). Our PCA identified 2 clusters, one involving 3 metabolites of Androstren - sterol/steroid pathways and the other comprising 2 gamma-glutamyl metabolites with pseudouridine. Furthermore, the loading plot (Figure 5) highlights patterns of features in different quadrants that suggest possible interacting pathways for future studies.

We finally interrogated the top 50 features from our ML model using pathway analysis in IPA. This highlighted more than 10 of the features most influential to our algorithm are significantly involved in systemic inflammatory pathways, pro-oxidative states via lipid peroxidation, and reactive oxygen species and nitrogen oxide radical generation. Canonical pathway analysis indicates a preponderance of features are represented in signalling, degradation, and biosynthesis pathways (Supplementary Figure 2).

### Model replication in QBB

We further tested the performance of our model in the QBB cohort. The test produced a mean absolute error of 14.69 mmHg, mean absolute percentage error of 13.39% and an R^2^ of 45.2% (Supplementary Figure 3). A SHAP plot of the top 50 features influencing SBP in the QBB cohort (Supplementary Figure 4) supports our findings from the TwinsUK cohort. In QBB, the top 50 features consisted of the 3 traditional risk factors, 4 biochemical measures, and 43 metabolites. Of these, 30 of the top 50 features identified in TwinsUK (Figure 3) were also identified in the top 50 features found to influence SBP in QBB (Table 2). Moreover, 8 of the top 10 features overlap between both cohorts, with age, BMI, dihomo-linolenate, urate, cis-4-decenoyl carnitine, and lactate being among the most influential features.

**Table 2:** Top 50 features between discovery and replication cohorts.

Feature order	Discovery: TwinsUK	Replication: QBB	Replicated*
1	Age	Age	Yes
2	BMI	BMI	Yes
3	Dihomo-linolenate (20:3n3 or n6)	Sex	Yes
4	Urate	Dihomo-linolenate	Yes
5	Cis-4-decenoyl carnitine	Urate	Yes
6	Lactate	Cis-4-decenoyl carnitine	Yes
7	Glucose	Lactate	No
8	Cortisol	Phenylacetylglutamine	Yes
9	Phosphate	Cortisol	No
10	Chloride	Histidine	Yes
11	Histidine	Gamma-glutamylphenylalanine	Yes
12	Glycine	Isobutrylcarnitine	No
13	HWESASXX*	1-Oleoyl-GPE	No
14	4-Androsten-3beta,17beta-diol monosulfate	4-Androsten-3beta,17beta-diol disulfate^1^*	No
15	1-Dihomo-linolenoyl-GPC (20:3n3 or 6)*	Chloride	No
16	N-Acetylglycine	N-Acetylglycine	Yes
17	4-Androsten-3beta,17beta-diol disulfate^1^*	Creatine	Yes
18	1-Arachidonoyl-GPE (20:4n6)*	Proline	Yes
19	Creatine	Phenol sulfate	Yes
20	Phenylacetylglutamine	Lysine	Yes
21	1-Oleoyl-GPE (18:1)	Gamma-glutamyltyrosine	Yes
22	Fructose	Pentadecanoate	No
23	4-Androsten-3beta,17beta-diol disulfate^2^*	1-Arachidnoyl-GPE	Yes
24	Calcium	Glycerol	Yes
25	Potassium	Carnitine	No
26	Sex	4-Androsten-3beta,17beta-diol disulfate^2^*	Yes
27	Dietary fat	3-Hydroxybutyrate	No
28	Alanine	Leucine	No
29	Hydroxyphenylacetic acid monosulfate	1,5-Anhydroglucitol	No
30	Dietary saturated fat	N-Acetylthreonine	No
31	2-Aminobutyrate	Malate	No
32	Dietary trans fat	Arginine	No
33	Gamma-glutamyltyrosine	1-Arachidnoyl-GPI	Yes
34	Lysine	Glutamine	Yes
35	Leucine	Pseudouridine	Yes
36	Phenol sulfate	Calcium	Yes
37	Isobutyrylcarnitine	Glycochenodeoxycholate	No
38	Glycerol	Cortisone	Yes
39	Gamma-glutamylphenylalanine	Glycocholate	Yes
40	Dietary sucrose	1-Palmitoleoyl-GPC	No
41	Adrenate (22:4n6)	Tryptophan	Yes
42	Glycocholate	Adrenate	Yes
43	Pseudouridine	Glycerophosphorylcholine	Yes
44	Carnitine	Gamma-glutamylleucine	Yes
45	N-Acetylthreonine	Octanoylcarnitine	Yes
46	Pyroglutamine*	Bilirubin (E,E)	No
47	Dietary selenium	Octadecanedioate	No
48	1-Arachidonoyl-GPI (20:4)*	1-Palmitoleoyl-GPE	Yes
49	N-Acetylcarnosine	Stearate	No
50	Biliverdin	Idolepropionate	No

*Feature replication column denotes if the feature in TwinsUK appeared in the top 50 features in QBB.

## Discussion

In this study, using a ML approach integrating traditional risk factors, biochemical measures, multi-omics, and dietary phenotypes to pull known risk factors together and understand how they interact, we were able to account for the multifactorial nature of BP and create a model that can explain 39·2% (± 4·5%) of the variance in SBP in TwinsUK and 45·2% (± 13·39%) in an ethnically diverse sample from QBB, overcoming the standard limitations of single omics and univariate models. We are also able to demonstrate the incremental value of each additional dimension included in the multivariable model indicating the considerable magnitude of effect of conventional variables and the smaller contributions from metabolomics and dietary data. Further interpretation of our model delineates the contributions of each feature involved in BP regulation while in context of one another. With the exception of age and BMI, the main features contributing to BP regulation were the metabolome, representing 35 of the top 50 features, 7 biochemical measures, including chloride, creatinine, calcium and potassium and dietary intake of 5 nutrients, including total fat, saturated fat, trans fat, sucrose and selenium (Figure 2). Moreover, a substantial proportion of these features overlap with the top 50 features influencing SBP in QBB highlighting the robustness of our analysis.

Although, other studies have attempted to integrate different types of data to predict BP,^14^^,^^15^ there are no other ML studies of BP, which have integrated data covering environmental, dietary, genetics, metabolites, biochemical, and clinical data to identify the top contributors.

### Metabolites

Our findings underscoring the involvement of the metabolome in BP, using an untargeted approach, is in-line with numerous metabolomic studies.^14^^,^^48^, ^49^, ^50^, ^51^, ^52^, ^53^, ^54^ Aside from clinical PRS, Drouard *et al.* report that metabolomic data contributed most to the predictive capacity of a ML model, with isoleucine, leucine, and several lipids performing best.^14^ Dietrich and colleagues^51^ investigated predictive metabolites for hypertension incidence using a targeted metabolomics approach in the EPIC cohort. Researchers identified 6 of the 127 metabolites to be most predictive for the development of hypertension (mean follow-up of 9·9 years). Among those, the up-regulation of both serine and glycine were associated with higher predicted hypertension-free survival. Likewise, our ML algorithm also identified glycine within the top 50 features involved in both SBP and DBP regulation and serine within the top features involved in DBP. Furthermore, SHAP analysis shows that higher levels of both glycine and serine correlated with lower predicted BP (Figure 3).

Figure 3 also shows that the metabolite 4-Androsten-3β,17β-diol disulfate 1, was the 17^th^ most influential feature involved in SBP, and it the 6^th^ most influential feature for DBP. Previously, our work has reported independent associations between this metabolite and both SBP and DBP (t test from linear regression models, SBP=1·82 (1·25; 2·38) P=3·95 × 10⁻^10^, DBP=1·25 (0·87;1·63) P=1·4 × 10^−10^),^52^ giving validation to our results. From our PCA analysis we see that 3 androstenediol metabolites, all of which were present in the top 50 features influencing SBP, separate from the other features to form a cluster (Figure 5). Androstenediols are intermediates in the biosynthesis of testosterone, a highly potent androgen.^55^ Multiple studies have suggested that androgens elicit positive effects on cardiovascular function, which is thought to be brought about by the mitigation of adipocyte and endothelial dysfunction.^56^ Though the molecular mechanisms behind this remain unclear, the leading hypothesis relates to immune and inflammatory responses via Nf-kB.^56^ Interestingly, canonical pathway analysis of the top 50 features influencing SBP in the IPA database suggests 13 of our top 50 features to be significantly linked to inflammatory response (Fisher's Exact test, P=1·56 × 10^−2,^) and 8 with inflammatory disease (Fisher's Exact test, P=1·3 × 10^−4^).

Our PCA analysis further shows gamma-glutamyltyrosine, gamma-glutamylphenylalanine, and pseudouridine separate to form a cluster. Gamma-glutamyl transferases (GGT) are enzymes responsible for the transfer of gamma-glutamyl to amino acids, and commonly used as biomarkers for alcohol intake and liver disease.^57^ GGT has also been previously linked with an increased risk of hypertension (RR = 1·2 (1·1: 1·31)).^57^ Interestingly, a determinant of elevated GGT is male sex, and in our PCA analysis conducting in a largely female sample (92·8%), we see no correlation between the gamma-glutamyl metabolite cluster and sex (Figure 5).

### Biochemical measures

Here, our ML algorithm signifies the role of multiple biochemical measures in BP regulation, including urate, and chloride (Figure 2).

Large epidemiology studies report inverse associations between serum chloride levels and mortality.^58^ In a longitudinal cross-sectional study, which was followed for at least 10-years, De Bacquer and Co.^59^ report an increased risk of all-cause, cardiovascular and non-cardiovascular mortality, independent of other classic risk factors. Researchers reported that the increased risk of cardiovascular mortality in women with low serum chloride more than doubled [RR = 2·16 (1·11: 4·22)].^59^ Similarly, Figure 3 shows that lower chloride levels predicted higher SBP in our model. Likewise, there are numerous large-scale epidemiology studies reporting associations between urate and hypertension^53^^,^^60^^,^^61^ or cardiovascular events.^62^^,^^63^ Here our ML algorithm implicates the role of urate for both SBP and DBP regulation. Moreover, urate featured 4^th^ within our SHAP plot (Figure 3), only behind age, BMI and dihomo-linolenate.

### Dietary intake

We report intake of total fat, saturated fat, and trans-fat, to greatly influence our SBP ML algorithm. Trans fat also featured highly within the SHAP analysis of features involved in DBP (Supplementary Figure 1). Contrary to some beliefs of a detrimental role of saturated fat intake in BP regulation, our SHAP plot (Figure 3) illustrates that those with greater dietary saturated fat intake had lower SBP. This is in keeping with our previous research, where we also show a significant negative correlation between saturated fat intake and SBP.^64^

Yet, the addition of dietary features to biochemical measures, traditional risk factors and metabolomic feature only marginally improved R^2^, however, this could be a result of diet already being proxied by the metabolomic data,^65^ or because of the subjectivity and innate high variability of dietary intake.

### Genomics

Despite including 891 recognised^25^ SNPs weighted by effect sizes into a PRS for BP, while in context of the other omic and biochemical measures, the PRS did not feature within the top 50. This supports the notion of a limited capacity for genomics to predict variation in BP.^66^^,^^67^ In contrast, although they aimed to measure the predictive utility of a ML model using different input feature to our study, Drouard and collaborators suggest a high predictive capacity for clinical PRS relating to body fat and known CVD risk factors, such as immune cell counts.^14^

Our work benefits from a large discovery cohort with an independent replication. It also benefits from a robust, clinically relevant ML algorithm which follows the current guidelines for ML studies^17^^,^^68^ and is able to integrate multiple omics and biochemical measures, and thereby explain a large proportion (39·2% ±4·5%) of the variance in SBP. A large proportion of features were identified in both cohorts, and some of those have also been previously established with BP, highlighting the robustness of our methods and results. However, our results should also be interpreted in the presence of a few caveats. First, our discovery sample was on average, middle-aged, and 92·8% were female and all were of white European descent, as such, we are unable to translate our findings to males. Nevertheless, we successfully replicated our results in an ethnically diverse replication sample, more than half of which were males. Second, dietary data was not available in QBB, which accounts for 5 (24%) of the top features that did not overlap between cohorts. Third, our data is cross-sectional, preventing any inferences of causation without further investigation. Fourth, dietary intake was measured using FFQs, which have numerous limitations, including reporting bias. Moreover, the duration between BP measurement and FFQ completion was on average 2·5 years, during this period habitual diet may have changed. However, any changes that may have occurred would have likely been marginal.^69^ Fifth, the algorithm used is unable to answer the question of reverse directionality, and any follow-on studies should seek to address this. Sixth, as this is the first ML model of BP to integrate this type of data, there are no other studies for direct comparisons or benchmarking. Seventh, we only had data to explore office BP. Office blood pressure has several limitations, including measurement error, and white coat effect.^70^ Other methods, such as continuous ambulatory BP can mitigate some of those limitations and is considered a more robust measurement of blood pressure. However, ambulatory BP was not available in TwinsUK. Finally, we imputed missing values across the entire dataset prior to cross-validation and this could result in data leakage into the test set, leading to potential overfitting.^71^ To minimize this possible bias from data leakage, we imputed the missing data in our study using an unsupervised imputation technique (KNN). The imputed data thus provided the ML model no insight into the later predictions.^72^ Moreover, recent studies observed negligible influence of conducting imputation on the whole dataset as compared to the training dataset on cross-validated performance.^71^^,^^72^ Hence, though we acknowledge this potential source of bias, as the top 50 features in our model had <3.8% missingness on average (Supplementary Table 3), we believe that the risk of data leakage and introduction of bias into the data with imputation prior to cross-validation is likely minimal. Future studies with even larger sample sizes should implement imputation by first imputing the training data, followed by application to the test set within each fold of cross-validation.

In conclusion, our study highlights the value of ML methods to integrate multimodal omics data to uncover the multifactorial contributors underlying the complexity of BP regulation. In doing so, we find that the most predictive features of BP are the traditional risk factors, metabolites and diet, while genetics (SNPs) does not appear to have a large role in this respect. This range of potential biomarkers for blood pressure should inform future studies. This set of follow-on studies should include validation in independent datasets, with a diverse sample, to infer clusters of factors that define population strata for clinical trials enhancing successes in identifying new treatments.

## Contributors

S. P., and C. M. conceived and designed the experiment; T. Q. B. ran the analysis; T. Q. B., and P. L verified the underlying data; P. L., S. P., and C. M., wrote the original manuscript. M. M., C. dT., P. C., and T. D. S., contributed methods/materials/analysis tools. All authors have read and approved the final version of the manuscript.

## Data sharing statement

The data used in this study are held by the department of Twin Research at King's College London. The data can be released to bona fide researchers using our normal procedures overseen by the Wellcome Trust and its guidelines as part of our core funding (https://twinsuk.ac.uk/resources-for-researchers/access-our-data/).

Access to Qatar Biobank data can be obtained through an established ISO-certified process by submitting a project request at https://www.qatarbiobank.org.qa/research/how-apply which is subject to approval by the Qatar Biobank IRB committee. The source code used in this study is freely available at: https://github.com/Tran031194/integrating_omics_BP.

## Declaration of interests

T. D. S is a co-founder and shareholder of Zoe Global. All other authors declare no competing financial interests.

## Appendix Supplementary materials

Image, application 1

Image, application 2
