# Supplementary material for: Machine learning integration of multimodal data identifies key features of blood pressure regulation
Source: eBioMedicine. 2022 Sep 6;84:104243. doi: 10.1016/j.ebiom.2022.104243 (PMC9463529; doi:10.1016/j.ebiom.2022.104243)
Supplement: Supplementary file 1 [file mmc1.docx]

**
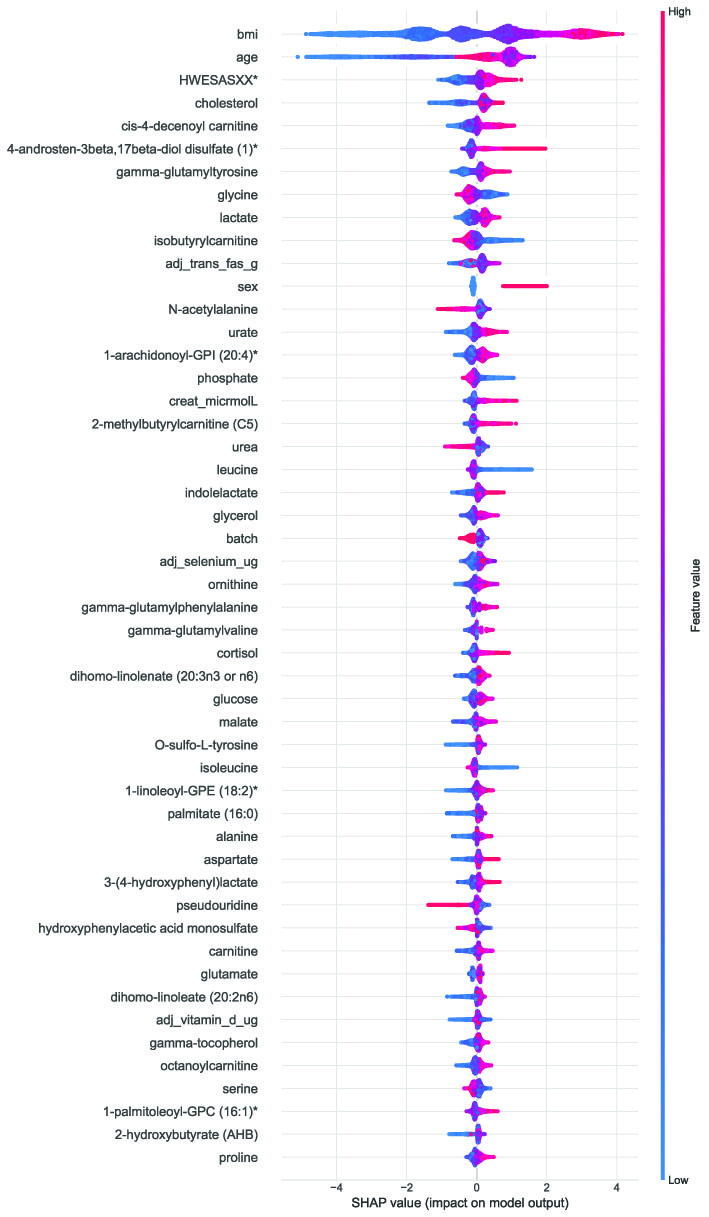
**

**Supplementary figure 1**. **SHAP plot of top 50 features influencing our model’s prediction of DBP.** Features are ranked in descending order based on their influence on our DBP model and the x-axis denotes SHAP values. Each dot represents an individual subject and is coloured according to the magnitude of the feature. Red depicts a higher feature value, and blue depicts a lower value. The horizontal location of a dot depicts whether it corresponds with a higher or lower prediction.


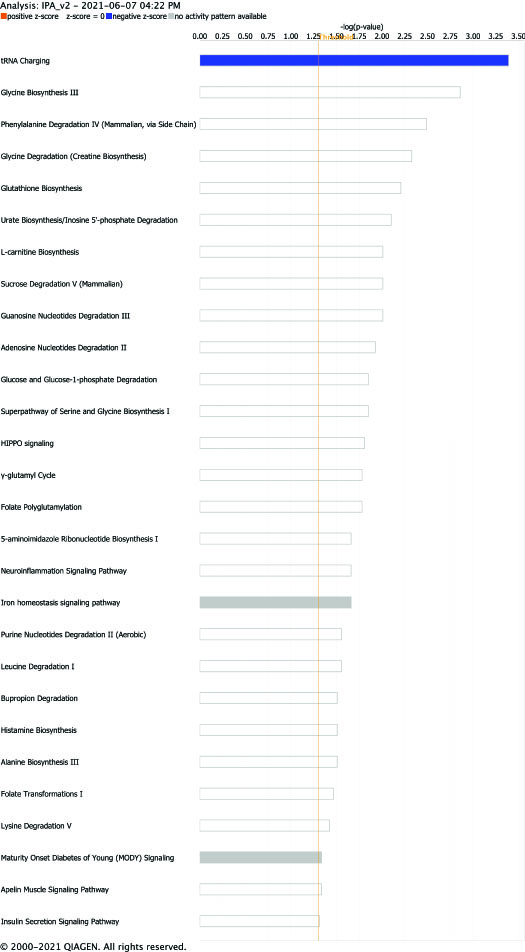


**Supplementary figure 2. Canonical pathways significantly associated with the top features from our model.** P< 0.05.

**
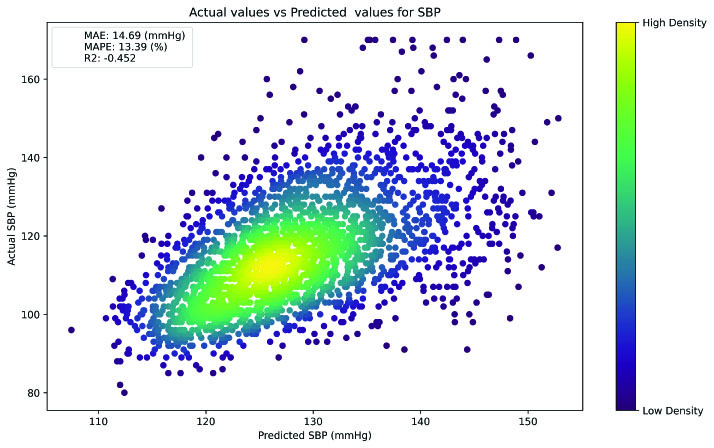
**

**Supplementary figure 3. Scatter plot of SBP values in QBB and predicted SBP**. Colour gradient scale depicts the density of participants within a particular region of the plot.

**
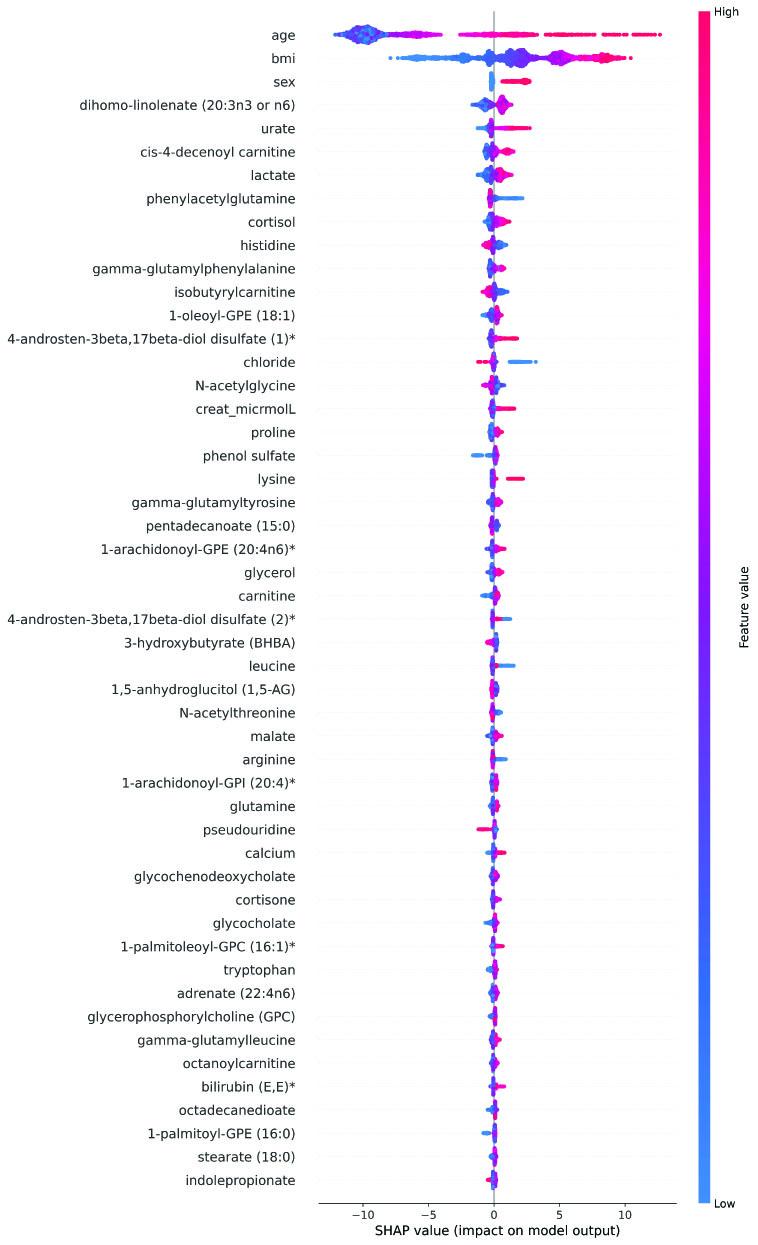
**

**Supplementary figure 4. SHAP plot of top 50 features influencing SBP in the QBB cohort.**
