# Supplementary material for: Machine learning integration of multimodal data identifies key features of blood pressure regulation
Source: eBioMedicine. 2022 Sep 6;84:104243. doi: 10.1016/j.ebiom.2022.104243 (PMC9463529; doi:10.1016/j.ebiom.2022.104243)
Supplement: Supplementary file 2 [file mmc2.docx]

**Supplementary Table 1. Hyperparameter tuning.** Parameters tested and the value selected for SBP model, female-only model, no FID model, and DBP model.

| Hyperparameter | Tested | SBP, female-only and no FID models | DBP model |
| --- | --- | --- | --- |
| colsample_bytree | 0.001 to 1, intervals of 0.001 | 0.943 | 0.277 |
| min_child_weight | 1 to 200, intervals of 1 | 197 | 197 |
| learning_rate | 0.001 to 1, intervals of 0.001 | 0.063 | 0.063 |
| n_estimators | 1 to 200, intervals of 1 | 137 | 137 |
| max_depth | 1 to 100, intervals of 1 | 6 | 6 |

**Supplementary Table 2. Model performance in our discovery cohort, TwinsUK.**

|  | Full model (n=4,863) | Female only model (n=4,513) | Full model without cluster id (n=4,863) | Age, sex, and BMI model (n=4,863) |
| --- | --- | --- | --- | --- |
| Mean absolute error (mmHg) | 11.32 (0.7) | 11.43 (0.9) | 11.45 (0.7) | 11.99 (0.4) |
| Mean absolute percentage error (%) | 8.92 (0.5) | 9.04 (0.7) | 9.06 (0.6) | 9.46 (0.2) |
| R^2^ (%) | 39.20 (4.5) | 33.45 (7.7) | 37.05 (5.6) | 33.00 (8.1) |

**Supplementary Table 3. Percentage of missingness within top 50 features implicated in BP regulation.**

| Feature order | Discovery: TwinsUK | % missingness |
| --- | --- | --- |
| 1 | Age | 0 |
| 2 | BMI | 0.37 |
| 3 | Dihomo-linolenate (20:3n3 or n6) | 0 |
| 4 | Urate | 0 |
| 5 | Cis-4-decenoyl carnitine | 19.12 |
| 6 | Lactate | 0 |
| 7 | Glucose | 0 |
| 8 | Cortisol | 0 |
| 9 | Phosphate | 0 |
| 10 | Chloride | 8.51 |
| 11 | Histidine | 0 |
| 12 | Glycine | 0 |
| 13 | HWESASXX* | 0 |
| 14 | 4-Androsten-3beta,17beta-diol monosulfate | 0 |
| 15 | 1-Dihomo-linolenoyl-GPC (20:3n3 or 6)* | 0 |
| 16 | N-Acetylglycine | 0 |
| 17 | 4-Androsten-3beta,17beta-diol disulfate (1)* | 19.12 |
| 18 | 1-Arachidonoyl-GPE (20:4n6)* | 0 |
| 19 | Creatine | 5.24 |
| 20 | Phenylacetylglutamine | 0 |
| 21 | 1-Oleoyl-GPE (18:1) | 0 |
| 22 | Fructose | 0 |
| 23 | 4-Androsten-3beta,17beta-diol disulfate (2)* | 19.12 |
| 24 | Calcium | 12.52 |
| 25 | Potassium | 12.79 |
| 26 | Sex | 0 |
| 27 | Dietary fat | 18.47 |
| 28 | Alanine | 0 |
| 29 | Hydroxyphenylacetic acid monosulfate | 0 |
| 30 | Dietary saturated fat | 18.47 |
| 31 | 2-Aminobutyrate | 0 |
| 32 | Dietary trans fat | 18.47 |
| 33 | Gamma-glutamyltyrosine | 0 |
| 34 | Lysine | 0 |
| 35 | Leucine | 0 |
| 36 | Phenol sulfate | 0 |
| 37 | Isobutyrylcarnitine | 0 |
| 38 | Glycerol | 0 |
| 39 | Gamma-glutamylphenylalanine | 0 |
| 40 | Dietary sucrose | 18.47 |
| 41 | Adrenate (22:4n6) | 0 |
| 42 | Glycocholate | 0 |
| 43 | Pseudouridine | 0 |
| 44 | Carnitine | 0 |
| 45 | N-Acetylthreonine | 0 |
| 46 | Pyroglutamine* | 0 |
| 47 | Dietary selenium | 18.47 |
| 48 | 1-Arachidonoyl-GPI (20:4)* | 0 |
| 49 | N-Acetylcarnosine | 2.47 |
| 50 | Biliverdin | 0 |
